# Intergenerational effects of CO_2_‐induced stream acidification in the Trinidadian guppy (*Poecilia reticulata*)

**DOI:** 10.1002/ece3.5761

**Published:** 2019-10-29

**Authors:** Hartley C. P. H. George, George Miles, James Bemrose, Amelia White, Matthew N. Bond, Tom C. Cameron

**Affiliations:** ^1^ School of Life Sciences University of Essex Colchester UK

**Keywords:** acidification, carbon dioxide, climate change, development, fishes, food availability, freshwater, growth rate, intergenerational effects, maternal acclimation, *Poecilia reticulata*

## Abstract

Rising atmospheric carbon dioxide levels are driving decreases in aquatic pH. As a result, there has been a surge in the number of studies examining the impact of acidification on aquatic fauna over the past decade. Thus far, both positive and negative impacts on the growth of fish have been reported, creating a disparity in results. Food availability and single‐generation exposure have been proposed as some of the reasons for these variable results, where unrealistically high food treatments lead to fish overcoming the energetic costs associated with acclimating to decreased pH. Likewise, exposure of fish to lower pH for only one generation may not capture the likely ecological response to acidification that wild populations might experience over two or more generations. Here we compare somatic growth rates of laboratory populations of the Trinidadian guppy (*Poecilia reticulata*) exposed to pH levels that represent the average and lowest levels observed in streams in its native range. Specifically, we test the role of maternal acclimation and resource availability on the response of freshwater fishes to acidification. Acidification had a negative impact on growth at more natural, low food treatments. With high food availability, fish whose mothers were acclimated to the acidified treatment showed no reduction in growth, compared to controls. Compensatory growth was observed in both control–acidified (maternal–natal environment) and acidified–control groups, where fish that did not experience intergenerational effects achieved the same size in response to acidification as those that did, after an initial period of stunted growth. These results suggest that future studies on the effects of shifting mean of aquatic pH on fishes should take account of intergenerational effects and compensatory growth, as otherwise effects of acidification may be overestimated.

## Graphical abstract

With concerns that decreasing marine and freshwater pH, fish development, and behavior will be negatively effected, we set out to test this in freshwater guppies and found that while stream acidification limits juvenile growth, both high resource availability and maternal exposure to acidified conditions can help fishes overcome previously observed negative effects of growth. A multi‐generation approach that considers plasticity and evolution is required to adequately assess the effects of environmental change on fishes.

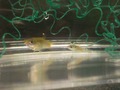

## 1 INTRODUCTION

Anthropogenic carbon dioxide (CO_2_) emissions, including the burning of fossil fuels and deforestation, are a key driver in both freshwater and ocean acidifications (Quay, Tilbrook, & Wong, 1992). In 2017, it is estimated that more than 36 billion tonnes of anthropogenic carbon dioxide was released into the earth's atmosphere (Le Quéré et al., 2018). Of this, between 65% and 80% will be absorbed by aquatic systems over the next 20–200 years, while the remainder will significantly contribute to global warming via the greenhouse effect (Archer et al., 2009).

Dissolution of CO_2_ into aquatic systems has a direct impact on pH through the formation of carbonic acid. The decreases in aquatic pH are predicted to continue with rising partial pressure CO (pCO_2_), posing a potential threat to aquatic fauna globally. Fish must continually maintain optimum internal pH, which generally differs from the pH of surrounding water (freshwater or marine) and hence leads to a gradient between internal and external pH (Leduc, Munday, Brown, & Ferrari, 2013). Changes in pH that deviate from what aquatic organisms are adapted to could result in increased maintenance costs for the individual (Baker & Brauner, 2012; Heuer & Grosell, 2016). Higher maintenance costs decrease energy availability for growth and reproduction and hence have the potential to reduce overall fitness.

Fish possess both chemical and physiological mechanisms to buffer fluctuations in environmental pH. The different mechanisms that cope with pH variability correspond to the length and severity of exposure that the fish must acclimate to (Kwong, Kumai, & Perry, 2014; Zahangir, Haque, Mostakim, & Islam, 2015). Fish have demonstrated they are capable of adapting to substantial pH changes when they occur over the long term, i.e., multiple generations (Tasoff & Johnson, 2019). While chronic responses to aquatic acidification via industrial pollution are well documented, e.g., sulfuric acid deposition, atmospheric CO_2_ concentration has increased by over 30% since 1900, yet no adverse effects in wild fish have been attributed to this rise (Etheridge et al., 1996; Tans & Keeling, 2018). However, current atmospheric CO_2_ levels have now exceeded any previous detectible level from the past 800,000 years (Lüthi et al., 2008). Thus, it is unknown whether fish are approaching the upper limit of their adaptive capacity. Previous experimental studies report complete pH compensation in both marine and freshwater fish over relatively short timescales, hours to days, postinduced acidosis (Larsen, Pörtner, & Jensen, 1997; Michaelidis, Spring, & Pörtner, 2007; Perry, 1982). This is managed through net acid secretion as well as efficient compensatory regulation of extracellular ${\text{HCO}}_{3}^{-}$ (Heuer & Grosell, 2014). Small, sudden decreases in pH are prevented by internal chemical buffering, which helps maintain stable hydrogen ion concentrations. However, there is building evidence that significant changes in CO_2_ concentration can have severe and, in some cases, fatal consequences for fish (Heuer & Grosell, 2014). Chemical buffering is also effective against more significant pH drops, over longer timescales, where acid–base ions are transferred via the gills (Heisler, 1984). Rising CO_2_ levels can also stimulate chemoreceptors which can increase gill ventilation when necessary, yielding greater transepithelial gas exchange, and direct removal of CO_2_ from the organism (Gilmour, 2001). Renal processes contribute negligibly to acid–base regulation in marine teleosts, but moderately in freshwater fish, and may help with long‐term survival in acidic environments, allowing direct excretion of ions (Claiborne, Edwards, & Morrison‐Shetlar, 2002; Claiborne, Walton, & Compton‐Mccullough, 1994; Perry & Gilmour, 2006). If the above systems cannot regulate internal pH sufficiently, for example due to sudden, large decreases in pH, then direct consequences for the fish will ensue. These are predominately reduced enzyme function, an imbalance in electrolytes, and hormone disturbance (Heisler, 1984; Heuer & Grosell, 2014).

Although increasingly well studied, the effects of CO_2_‐induced acidification on fish life history are not well understood. The reported impacts of acidification due to exposure of increased CO_2_ levels on fish are broad and highly variable, with reports of both enhanced and reduced growth rates, as well as no effect at all (Baumann, Talmage, & Gobler, 2012; Munday, Gagliano, Donelson, Dixson, & Thorrold, 2011; Rossi et al., 2015; Sswat, Stiasny, Jutfelt, Riebesell, & Clemmesen, 2018). In addition, diverse responses to the same acidification treatments have been reported within single experiments, implying a varying level of phenotypic plasticity within species. In one such study with marine fish, the settlement‐stage offspring of some *Amphiprion percula* pairs were larger at increased pCO_2_ treatments, compared to control, while the offspring from other pairs were smaller (Munday, Donelson, Dixson, & Endo, 2009). A meta‐analysis concluded that ocean acidification has an overall positive effect on fish growth (Kroeker, Kordas, Crim, & Singh, 2010), with more recent studies continuing to find similar results (Rossi et al., 2015). Similarly, both enhanced aerobic scope and increased routine metabolic rate have been reported in fish exposed to near‐future CO_2_ levels (Miller, Watson, Donelson, McCormick, & Munday, 2012; Rummer et al., 2013). Therefore, there is a growing consensus on the response of fishes within acidification studies, but the focus of these studies is largely on tropical or temperate marine species.

There is a need for focus on the effects of contemporary and anthropogenic climate change of freshwater systems and fishes, especially due to the long‐standing crisis in freshwater biodiversity (Hannan & Rummer, 2018; Reid et al., 2018). The original research focus on freshwater acidification on aquatic life arose from rising sulfuric acid concentrations from acid rain during the 1970s and 1980s, associated with industrial practices (Schindler, 1988). Significant fish mortality followed and in less than 20 years, for example, the number of lakes devoid of fish in southern Norway doubled as a result of pH decreases (Henriksen, Lien, Rosseland, Traaen, & Sevaldrud, 1989). Although the introduction of either sulfuric or carbonic acids to freshwater results in decreased pH, the effects on freshwater organisms are incomparable. Sulfuric acid is a strong acid, achieving close to 100% ionization when in solution, conversely, carbonic acid is a weak acid and only partly dissociates (Pitzer, 1937). Moreover, acid rain led to significant acid deposition over relatively short timescales, in some cases leading to abrupt and widespread fish mortality (Leivestad & Muniz, 1976). This differs to CO_2_‐induced acidification, which is occurring over greater timescales, and so, it is not appropriate to make direct comparisons between the effects of CO_2_ and sulfuric acid (Lüthi et al., 2008). There is therefore a genuine knowledge gap on how freshwater fishes will respond to reduced CO_2_‐induced pH over multiple generations.

Under natural conditions, maternal effects can make transient, resource‐based, and epigenetic changes to their offspring (Bonduriansky & Day, 2009; Kirkpatrick & Lande, 1989). These intergenerational effects have been documented in both marine and freshwater fish species in response to changes in the parental environment (Furness, Lee, & Reznick, 2015; Leips, Richardson, Rodd, & Travis, 2009; Stiasny et al., 2018). If intergenerational effects allow for the preacclimation of freshwater offspring to acidified environments, thereby reducing the metabolic demand on juveniles and allowing them to compensate for any effects on growth or metabolism, adaptation to a high‐CO_2_ environment may be much faster than expected and have minimal effects on the status of freshwater fish populations. However when under high metabolic demand, for example during the larval life stage, the increased energetic cost associated with compensatory mechanisms may lead to energetic deficit in acidified fish (Stiasny et al., 2018). This effect may be exacerbated when feeding is limited, as is often the case for wild fish larvae (MacKenzie, Leggett, & Peters, 1990). This has been demonstrated in juvenile blue mussels (*Mytilus edulis*), in both the laboratory and field, where high food availability offsets the effects of CO_2_‐induced acidification on growth (Thomsen, Casties, Pansch, Körtzinger, & Melzner, 2013). This indicates that increased maintenance costs, as a result of high pCO_2_, are linked to a reduced investment in growth. We therefore suggest that contradictory results in life history trait responses to acidification in fishes may be partly explained through different nutritional inputs used in different laboratory‐based experimental studies. In the laboratory environment, it is commonplace to provide ad libitum food levels, so not to compromise survival (Gordon, Kaiser, Britz, & Hecht, 2000). The practical implications of controlling the fixed food rations, especially if using live food, are laborious, but not doing so can lead to food‐rich conditions that are unrepresentative of wild systems and results that are difficult to compare between laboratories. In addition, ensuring each animal receives a similar food level involves limiting competition, which may mean fish must be housed individually. Recently, including the interaction between food level and CO_2_ treatment has become more common in studies examining larval growth under CO_2_‐induced acidification, yet the interaction has not yet been examined in a freshwater environment (Gobler, Merlo, Morrell, & Griffith, 2018; Hurst, Laurel, Hanneman, Haines, & Ottmar, 2017; Sswat, Stiasny, Taucher, et al., 2018; Stiasny et al., 2018, 2019).

Here we use the model system of Trinidadian guppies (*Poecilia reticulata*), originating from low‐predation streams on Trinidad (original collection c2009), to investigate how juvenile somatic growth rate responds to food availability and acidification in a multifactorial experiment. Low‐predation streams are characterized by high intraspecific competition, owing to the lack of predation, which leads to high levels of cannibalism within the population. Hence, low‐predation *P. reticulata* generally show fast initial growth until they reach a size where the likelihood of cannibalism will be minimized and the individual is more competitive within the population. It is the initial growth rate that is of particular interest here, as reductions would likely lead to significantly increased mortality in wild populations (Reznick, Butler, & Rodd, 2001; Reznick & Endler, 1982). We expect fish in more acidified treatments to grow more slowly under lower food treatments, but not in higher ad libitum food treatments and fish born into novel environments to grow more slowly than those born into environments that their mothers were acclimated to at preparturition.

## 2 MATERIALS AND METHODS

### 2.1 Study organism

Guppies (*P. reticulata*) used for this study were taken from laboratory stock populations, originating from Quare stream, Trinidad. *P. reticulata* are live bearing and feature high reproduction and developmental rates, making them suitable for studying intergenerational responses of growth to environmental change. Original stock populations were maintained at a standardized pH of ~8.40, 2 ppt salinity, and a temperature of 26.5°C, for multiple generations on ZM‐400 granulated fish food (ZM Systems). All fish utilized in this study come from this original stock population.

### 2.2 Experimental design

A multifactorial design was implemented to examine the impact of acidification on somatic growth rate at low and high (near ad libitum) food levels in both the presence and absence of maternal acclimation to higher CO_2_‐acidified conditions.

#### 2.2.1 Experiment 1: acidification

Twenty‐four juvenile *P. reticulata*, of less than one week of age, were taken from control pH conditions in stock tanks split across two rearing environments: control source tank–control rearing versus control source–acidified rearing. To control the environmental food availability on the response to environmental stress, the growth of each juvenile fish in each maternal‐rearing environment was examined at both high and low food availability. This results in six replicate fish per treatment combination, limited by availability of day‐old fish at that time.

#### 2.2.2 Experiment 2: intergenerational effects

**Figure 1:**
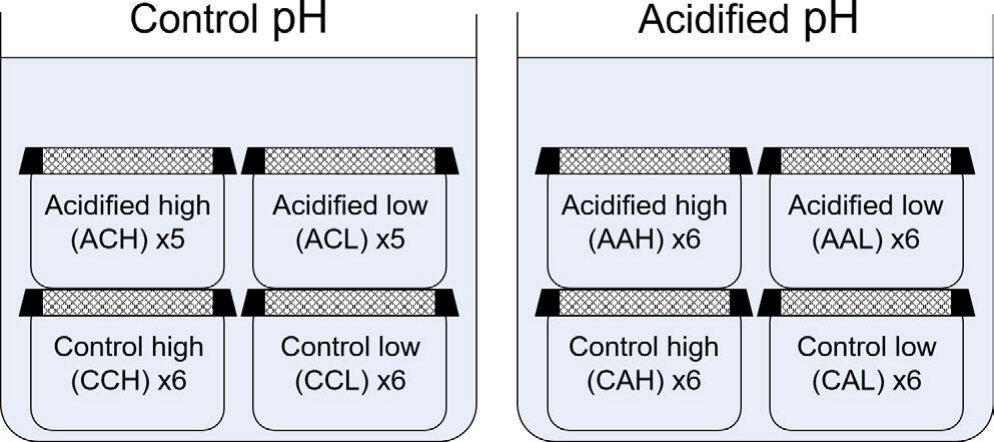
Schematic displaying the setup for Experiment 2. “Acidified” or “control” labels in each box refer to the maternal environment at parturition, and “high” or “low” refers to the food level the juvenile received. Each of the 46 (48 minus 2 mortalities) fish was kept in isolation in a flow through container, within a water bath at either control or acidified pH environment

To determine the impact of intergenerational effects through maternal acclimation to acidified conditions as described in Experiment 1, recently mated female guppies from the original stock tanks in Experiment 1 were placed in acidified conditions for the duration of their pregnancies. The F_1_ offspring from these females were divided into control and acidified treatments to give new maternal–current treatments: acidified–acidified and acidified–control to compare to juvenile growth rates being collected in Experiment 1. As some of these acidified mothers aborted dead offspring, the young from two females with larger litters was used more than others. The experiment started with *n* = 6 newborn juveniles for all pH–food availability treatments; however, one individual died in each of the acidified–control groups at both high and low food levels during the experiment. As a result, the replication dropped to *n* = 5 for ACH and ACL (Figure 1). Due to the limited time available for experimentation, it was not possible to mate nongravid females and hence ensure that pregnancies began once the fish were in the acidified treatment. Instead, any effects of maternal acclimation would solely occur by transfer through the umbilical cord or other unspecified mechanisms in pregnant females.

Both Experiments 1 (8 weeks duration) and 2 (5 weeks duration) were conducted between May and September in 2017. Method development for the controlled food growth assays and maintaining acidified laboratory populations of guppies occurred from 2015 to 2017.

### 2.3 Experimental setup

To investigate the effects of CO_2_‐induced acidification on potential intergenerational responses in our model species, we exposed guppies to the minimum pH of wild streams in Trinidad where guppies can be found. An initial acidified treatment of pH 6.20 led to higher mortality than expected. Consequently, the pH was increased to 6.50 and was chosen for the acidified treatment; this represents a pH more commonly recorded in Trinidadian streams where *P. reticulata* are known to live and lead to 100% survival (Enviornmental Management Authority, 1998). Although this pH is significantly lower than that used for a majority of ocean acidification studies, it is commonplace to use such levels in freshwater experiments (Midway, Hasler, Wagner, & Suski, 2017; Tix, Hasler, Sullivan, Jeffrey, & Suski, 2017). Moreover, the quantity of CO_2_ within freshwater systems fluctuates much more than in saltwater due to reduced buffering capacity and volume of freshwater bodies. Freshwaters, lotic systems in particular, are known to absorb CO_2_ to such a degree that they become supersaturated, achieving CO_2_ pressures one to two orders of magnitude higher than atmospheric levels (Hasler, Butman, Jeffrey, & Suski, 2016; Richey, Brock, Naiman, Wissmar, & Stallard, 1980; Telmer & Veizer, 1999). This can result in pH reductions of ~2.0 occurring over just 30 years (Andersson & Olsson, 1985). This was contrasted to the control treatment pH of 8.2 that laboratory stock populations had become adapted to over several generations since their initial collection in 2006–2009 (Nilsson, Lundbäck, Postavnicheva‐Harri, & Persson, 2011).

pH for the acidified treatment was maintained by an Aqua Medic^®^ CO_2_ computer coupled with a solenoid valve and multi‐pH probe (Hasler, Midway, et al., 2016; Kates, Dennis, Noatch, & Suski, 2012; Midway et al., 2017). This was set to a threshold of 6.52, above which the solenoid valve would open and CO_2_ gas would bubble through the treatment tank until a pH of 6.50 was achieved. Occasionally, a delay occurred between the pH computer detecting the pH and the dissolution of CO_2_, leading to a mild overshoot in acidification and achieving values of 6.47. Therefore, the acidified treatment is described as 6.50 (±0.03) and the control as 8.2 (±0.1). The pH computer was calibrated in line with manufacturer instructions to ensure pH readings were consistently accurate. The pH was also regularly checked with both a handheld pH meter and color test kits (API pH test kit) to validate readings. Control treatment pH was maintained through regular 20% weekly water changes using reverse osmosis water buffered with Salifert^®^ KH and pH buffer with regular manual check of pH, while unbuffered water was used for the acidified treatment. All tanks were held at a temperature of 26.5°C (±0.2), and water quality was maintained via internal powerhead sponge filters.

Fish were housed individually in one liter Exo Terra^®^ containers, submerged within either control or acidified tanks (see schematic in Figure 1). This ensured equal water parameters for each fish within each treatment, while also allowing for the tracking of individual growth rate in isolated fish, but is open to critique for not having separate header/source tanks for each fish. The removal of competition guaranteed that the intended food levels were available only to each individual. Regular water changes and removal of detritus also allowed for accurate responses to food level.

### 2.4 Feeding

Fish were fed a homogenous liquid fry food called Liquifry Interpet^®^ liquifry No. 2 (dry weight analysis: protein 34.4%, oil 13.5%, fiber 1.0%, ash 5.6%) for a period of 8 weeks. This was chosen as, unlike pellet or flake food, it can be easily and accurately measured and remained within fish containers after feeding, ensuring the entire ration was received by an individual fish. Feeding levels, as used in the following experiments, were established as low (0.1 ml) and high (0.3 ml) volumes by a previous pilot study (Bemrose, 2017). Fish were fed either high or low food per capita per day with a one day absence of feeding each week.

### 2.5 Measurements

**Figure 2:**
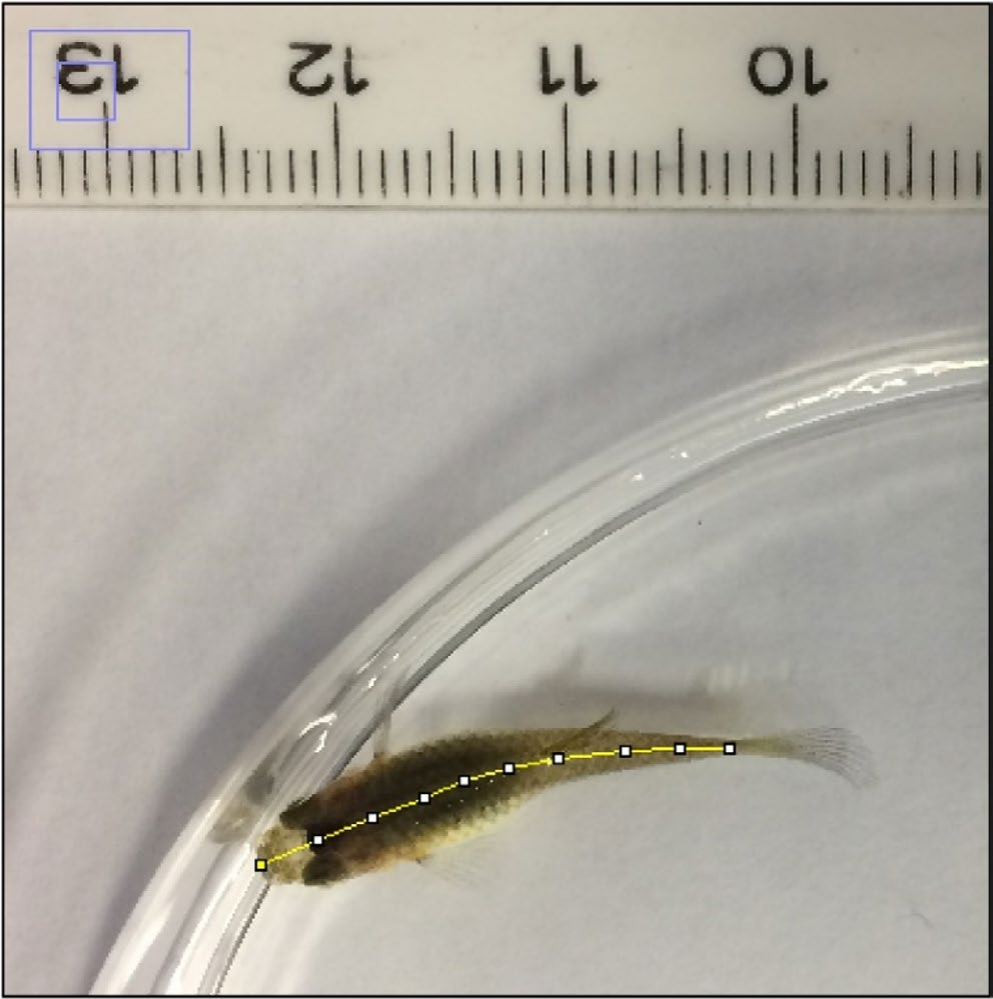
Segmented line drawn along spinal cord of fish, from tip of snout to caudal peduncle, to accurately measure the standard length of each fish using ImageJ. Each image captured with scale reference. Credit: Hartley George

In both experiments, fish standard length (SL) was measured once weekly as a proxy for somatic growth. Other measurements were not taken to minimize handling which we have found can affect the results of growth experiments. Each fish was removed from its container and individually photographed adjacent to a scale bar with a tripod‐mounted Logitech^®^ HD PRO C920 webcam. Images were subsequently analyzed with ImageJ to a precision of 0.001 mm, by drawing a segmented line along the spinal cord of the fish from the tip of the snout to the caudal peduncle (Figure 2). SL was chosen as the sole measure of fish size as SL is strongly correlated with somatic growth and biomass in *P. reticulata* (Barlow, 1992). This also allowed us to minimize handling time of individual fish.

### 2.6 Statistical analysis

All plots and statistical analysis were carried out in R (R Core Team, 2016) and using the *ggplot2* package (Wickham, 2009). To take into account the repeated measures between the multiple observations of each fish, a linear mixed effects model of the relationship between fish SL, food level, and acidification treatment was obtained using *lme4* where fish identify was a random term (Bates, Mächler, Bolker, & Walker, 2015). Note that in some plots for ease of interpretation these changes are shown as percentages (e.g., Figure 4), but the analysis was on the raw body size measurements. Age at measurement, food level, and acidification treatment were fixed effects with full interaction terms. Residual plots were visually inspected, and no deviations from normality or homoscedasticity were of concern. Likelihood ratio tests were used to test for significance of the retention of each factor and its interactions. Deletion of nonsignificant terms from a maximal model took place, until the minimum adequate model was determined. *Z* tests with Tukey adjusted multiple comparisons were carried out on the model to determine differences between levels for each factor following model simplification.

## 3 RESULTS

### 3.1 Experiment 1: acidification

**Figure 3:**
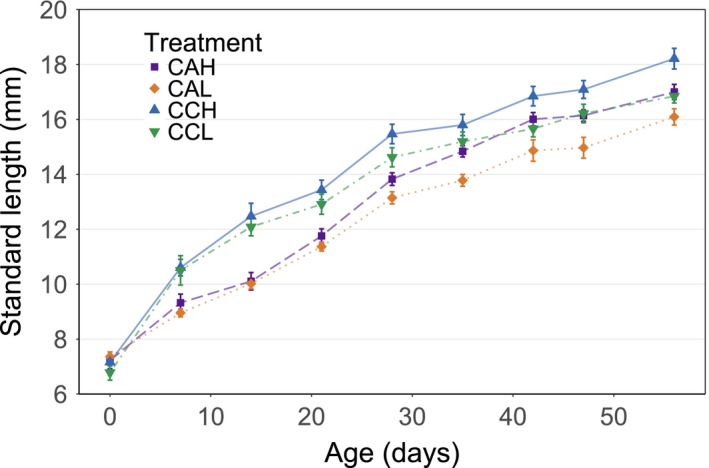
Effect of rearing environment, at both high and low food availability, on the growth of *Poecilia reticulata*. Error bars show ±1 *SE*. CAH, acidified high food; CAL, acidified low food; CCH, control high food; CCL, control low food

Acidification led to an overall reduction in fish SL by 1.17 mm (±0.24 SE), compared to control fish (*X* ^2^_1,7_ = 17.81, *p* < .001). This was true for both high (*Z*_1,7_ = 3.85, *p* < .005) and low (*Z*_1,7_ = 3.63, *p* < .01) food availability. The food treatments were indistinguishable until day 28, at which point the effect of the differing food levels became apparent (Figure 3). As such, high food availability had a greater effect on fish size toward the end of the experiment, meaning there were interacting effects of food levels and ages of fish (*X* ^2^_1,8_ = 8.47, *p* < .004). The average SL of fish per time step in the low food groups was 0.02 mm (±0.01) smaller, compared to high food. By the end of the experiment, the acidified high (AH) treatment was approximately the same length as control low (CL) (17.00 ± 0.67 mm, 16.8 ± 0.60, respectively).

### 3.2 Experiment 2: intergenerational effects

**Figure 4:**
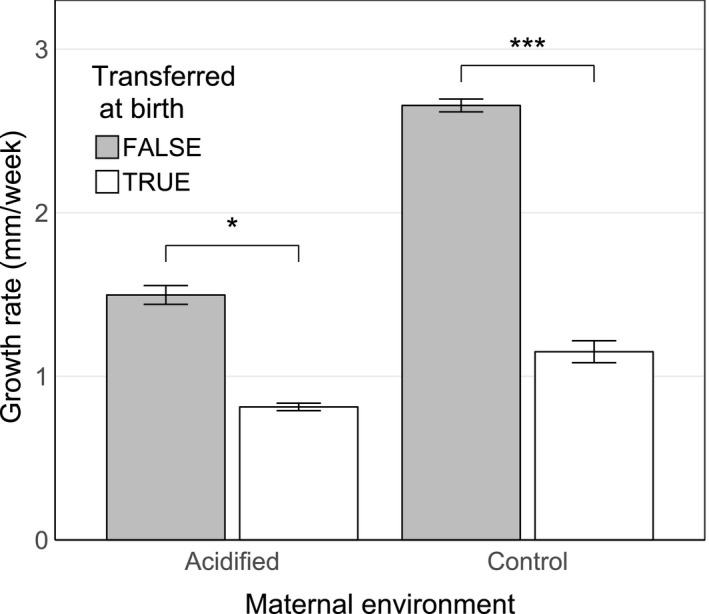
Somatic growth rate during first week of life in fish that were raised in their maternal environment (acidified–acidified & control–control) versus fish that were transferred into a different environment at birth (acidified–control & control–acidified). *p < .05; ***p < .001. Error bars are one standard error of the mean

**Figure 5:**
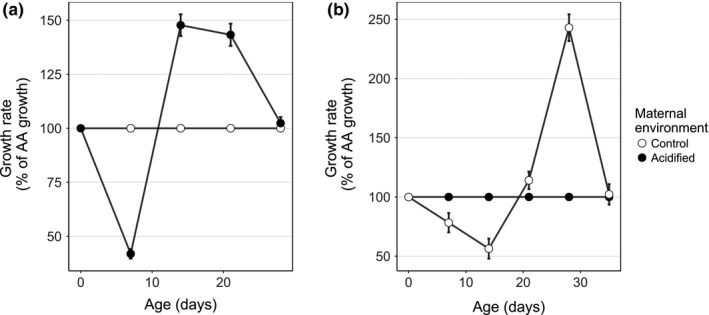
Growth rate for fish (a) reared in acidified conditions and (b) reared in control conditions which occurred between measurements, expressed as a percentage of the control growth rate, displaying compensatory growth when under high food availability. The control group is that which experienced intergenerational effects. After Mortensen and Damsgård (1993)

Growth in the acidified–acidified group was not significantly lower than in the control–control group, for either high or low food (*Z*_1,8_ = 1.38, *p* = .87; *Z*_1,8_ = 1.54, *p* = .79, respectively). For both high and low food availability, overall growth did not differ between either control–acidified and acidified–acidified groups (*Z*_1,8_ = 2.30, *p* = .29; *Z*_1,8_ = 1.93, *p* = .53) or acidified–control and control–control groups (*Z*_1,8_ = −0.03, *p* = 1.00; *Z*_1,8_ = 1.49, *p* = .81). However, there was an interaction between age and maternal environment for both treatments (acidified: *X* ^2^_1,8_ = 24.598, *p* < .001; control: *X* ^2^_1,8_ = 28.492, *p* < .001), implying the effect of maternal environment changed with time. At the outset, fish reared in their maternal environment featured higher initial growth rate than those born into that same environment, but then transferred to a different environment (Figure 4). Acidified–acidified showed greater initial growth than acidified–control (*Z*_1,11_ = 2.65, *p* < .04) and control–control showed greater initial growth than control–acidified (*Z*_1,9_ = 6.12, *p* < .001). However, after a short period of decreased growth rate (~14 days), the high food treatments, and to a lesser extent the low food treatments, underwent a period of enhanced growth, allowing fish that did not experience intergenerational effects to achieve a similar size as fish born into their maternal environment (Figure 5).

## 4 DISCUSSION

We have demonstrated that acidification has a negative effect on juvenile growth under low food availability. However, high food availability did not fully compensate for the effects of acidification on fish growth rate as has been demonstrated elsewhere (Thomsen et al., 2013). Instead, a negative effect of acidification was found for both food levels. The separation between high and low food levels was not apparent until approximately day 21; after this time, the high food treatments began to show more rapid growth. This was most likely because the low food level was not limiting until this point in the juvenile organism's development due to low initial energetic requirements. Nevertheless, the low food treatment was implemented to mimic a food level typical of the wild, which it achieved. Growth rates were similar to those measured in wild *P. reticulata* originating from low‐predation streams, approximately 1 mm growth over a 12‐day period in 12–14 mm fish (Reznick et al., 2001). However, elevated pCO_2_ has been shown to decrease the nutritional quality of freshwater phytoplankton as well as alter plankton community structure (Hasler, Butman, et al., 2016). It may therefore be argued that future climate conditions will lead to nutritional availability lower than that used in this study.

It was proposed that the reason for reports of acidification having no effect on, or increasing, growth rate of juvenile fishes was due to the ad libitum food levels provided in a majority of studies masking the increased energetic cost associated with acclimating to an acidified environment, such as in several studies on juvenile marine fishes (Munday et al., 2009; Rossi et al., 2015). This would imply an interaction between acidification and food availability, as high food leads to more growth in fish raised under acidified conditions compared to control. At the controlled food levels used in this study, no statistically significant interaction between acidification and food level was found. This may be because the high food level was not truly ad libitum, as in other studies. Yet, under high food availability, acidified fish still achieved the same size as fish reared in control pH under low food availability. Hence, the high energy availability allowed fish to better deal with the effects of increased energy consumption that occurs as a result of utilizing compensatory mechanisms such as maintaining extracellular HCO_3_^−^ concentrations (Ishimatsu, Hayashi, & Kikkawa, 2008). We conclude that high food availability does allow guppies to partially offset the energetic costs of adjusting to acidification.

### 4.1 Intergenerational effects

Fish born of mothers reared in control conditions but themselves reared in acidified conditions (control–acidified) grew more slowly than control–control fish, the acidified–acidified fish grew only marginally, and not significantly, slower than control–control fish. Maternal environment therefore had a small but significant impact on mediating the effects of acidification on growth. A very similar result was found in the marine anemonefish *Amphiprion melanopus* (Miller et al., 2012). Furthermore, the significant interaction we have discovered between maternal environment and fish age implies maternal environment affected growth differently at different ages or life stages. Maternal environments have been proposed to have the most effect on very early offspring life stages as it is during this critical period that preacclimated juvenile physiology yielded the most benefit from parental effects (Allen, Buckley, & Marshall, 2007; Benton, St Clair, & Plaistow, 2008). In this study, it was demonstrated that manipulating the juvenile environment so that it differed from the maternal environment leads to a decrease in growth rate during that first week of life. This was not reported by Miller et al. (2012), as body length was only measured once, not repeatedly, demonstrating the value of studying growth rates through time and not only size at age or maturity. Transgenerational epigenetics are transient modifications that alter offspring phenotype and can occur as a result of changes to the parental environment. They have been shown to be important in preacclimating juveniles to their environment, when the maternal environment is changed prior to, or during, gestation (Szyf, 2015). In live‐bearing fishes, such as guppies, the mechanisms that lead to transgenerational epigenetic changes are still unclear (Le Roy, Loughland, & Seebacher, 2017), and given the relatively short‐term maternal environment exposure of our mothers in this study, it is not possible or appropriate to infer any role of epigenetics.

Overall, maternal pH acclimation appears to reduce energetic costs that otherwise limit juvenile investment into somatic growth. In this study, adult females were provided with high food levels to minimize energetic stress when acclimated to the acidified treatment. However if, for example, maternal food availability was limited, then the increased energetic cost for the mother may lead to her constraining energetic input into reproduction (Miller et al., 2012). Consequences could include decreased fry provisioning, with a resultant decrease in survival.

During this study, 12 pregnant female guppies were acclimated to the acidified treatment and provided with high‐quality food in an attempt to determine how maternal investment varied with acidification. However, at least two fish aborted dead, underdeveloped fry, and another aborted a morphologically compromised fry. None such observations occurred under the control pH, and similar observations have been observed in our acidified stock tanks and reported elsewhere (Baumann et al., 2012). It is common for animals to decrease reproductive efforts when food is limited, and viviparous organisms are known to reabsorb embryos. It is therefore possible that the increased energetic cost associated with acclimating to an acidified environment could lead to similar effects (Metcalfe & Monaghan, 2001). This finding challenges the claims that exposure of adult fish to near‐future CO_2_ does not have significant energetic costs (Ishimatsu et al., 2008). However, CO_2_‐induced acidification is a gradual process occurring continuously over multiple generations in wild fish, which could give rise to much longer periods of transgenerational acclimation in the wild than we were able to simulate here (Stiasny et al., 2018).

### 4.2 Compensatory growth

The acidified–acidified and control–control groups were preacclimated to the acidified and control environments, respectively, whereas the control–acidified and acidified–control were not. However, acidified–control high food and control–acidified high food, and to a lesser extent acidified–control and control–acidified low food treatments, attained the same size as their respective preacclimated groups by day 28, following a period of stunted growth. The suppressed growth rate that occurred in nonacclimated fish ended by day 14, after which point growth rate in the high food treatment increased substantially. Here we propose that juvenile fish grow more slowly during the first 14 days because they are investing energy in pH compensatory mechanisms, including altering their physiology to suit the pH of their new environment, rather than in somatic growth. This leads to a disruption between chronological and developmental age, whereby individuals are smaller in length than what would usually be determined by their chronological age (Wilson & Osbourn, 1960). Postacclimation, acidified–control high food and control–acidified high food made use of the high food availability and grew at a rate greater than what was achieved by the preacclimated groups (CCH and AAH), to achieve the same size as the preacclimated by day 28, a form of compensatory growth.

Compensatory growth traditionally defines a period of accelerated somatic growth, to a level which exceeds that of routine growth, as a result of an increase in energetic resources, following a period of restricted resources and consequential growth retardation (Auer, Arendt, Chandramouli, & Reznick, 2010; Metcalfe & Monaghan, 2001; Wilson & Osbourn, 1960). Classic experimental studies of compensatory growth in freshwater fish include a period in an environment deliberately manipulated to decrease growth, such as low temperature, before being moved to a control environment and observing an increase in growth, greater than what the control ever showed (Mortensen & Damsgård, 1993; Nicieza & Metcalfe, 1997). Here, the accelerated growth occurred not after direct manipulation of the treatment, but instead after fish became large enough to compensate for the effects of the treatment.

In anadromous fish, such as salmon, compensatory growth occurs because fish must achieve a particular size before smoltification, and if they do not, then they must delay their migration, at significant cost to the organism (Mortensen & Damsgård, 1993). For low‐predation guppies, the pressure to achieve a certain size may be related to predation, specifically risk of cannibalism, and becoming more competitive within the population. Thus, if juveniles do not accelerate their growth, they would likely suffer increased mortality (Reznick et al., 2001). Although compensatory growth serves a direct purpose, to negate the immediate impact of smaller body sizes, it has previously been shown to have lasting detrimental effects (De Block & Stoks, 2008; Johnsson & Bohlin, 2006; Metcalfe & Monaghan, 2001). In particular, compensatory growth has been demonstrated to reduce litter size in *P. reticulata*, with a resultant 20% decrease in offspring production (Auer et al., 2010). Studies which use fish that have not experienced intergenerational effects, and therefore show reduced reproduction, may misinterpret this result as acidification, leading to a reduction in recruitment.

## 5 CONCLUSION

This study was designed to assess the impact of CO_2_‐induced aquatic acidification on the growth of a freshwater fish. Experimental pH levels were chosen that are already found in streams native to the study organism. It was demonstrated that exposure to elevated CO_2_ reduces growth in wild fish that are naturally food limited, but this may be somewhat mediated by maternal acclimation. Replication was low in this study, and we encourage others to repeat our work to help find how general it may be. By not allowing for intergenerational effects, a majority of studies are missing an important factor in their conclusions of the effects of future climate conditions and may even be overestimating certain impacts. The detection of compensatory growth in control–acidified treatments is further evidence to allow for intergenerational effects in future studies. In a past protocol, set out in an attempt to standardize ocean acidification studies, it is advised to replicate natural food levels as best as possible to ensure results from laboratory studies are relatable to the wild (Riebesell, Fabry, Hansson, & Gattuso, 2011). However, a majority of studies still use ad libitum food levels. The logistics associated with limiting feeding of larval marine fish are challenging, and significantly lower survival rates are to be expected, as in the wild (Dahlberg, 1979; Gordon et al., 2000). However, the use of ad libitum food levels is likely masking effects that would otherwise be detected during the CO_2_‐sensitive larval stage (Sayer, Reader, & Dalziel, 1993).

Owing to the aforementioned variability in the effects of acidification on different fish species, it is now paramount to attempt to replicate these results in other fish species. This will help gain a truer understanding of what drives changes in fish life history traits as a result of exposure to elevated CO_2_. Research into the effects of CO_2_‐induced acidification on freshwater fish is gaining momentum. These results, with regard to both food availability and maternal environment, should be considered in the design of future studies if progress is to be made in determining the true effects of freshwater CO_2_ acidification.

## AUTHOR CONTRIBUTIONS

HG and TCC developed the initial concept and with MNB developed the experiment. HG conducted the experiments and initial analysis. GM, JB, and AW all contributed to the feeding and growth measurement method development used in this experiment, without which the work would not have been possible. HG wrote the initial manuscript, and MNB and TCC contributed to revisions before submission.

## Data Availability

All data used in this study are available in the University of Essex Research Data repository (https://dx.doi.org/10.5526/ERDR-00000117).
